# Prognostic nomograms integrating preoperative serum lipid derivative and systemic inflammatory marker of patients with non-metastatic colorectal cancer undergoing curative resection

**DOI:** 10.3389/fonc.2023.1100820

**Published:** 2023-03-09

**Authors:** Dimei Huang, Shaochu Zheng, Fang Huang, Jingyu Chen, Yuexiang Zhang, Yusha Chen, Bixun Li

**Affiliations:** ^1^ Department of General Internal Medicine, Affiliated Tumor Hospital of Guangxi Medical University, Nanning, China; ^2^ Department of Haematology/Oncology and Paediatric Oncology, Affiliated Tumor Hospital of Guangxi Medical University, Nanning, China; ^3^ Department of Oncology, Affiliated Changzhi People’s Hospital of Changzhi Medical College, Changzhi, China; ^4^ Department of Oncology, Affiliated Hospital of Youjiang Medical University for Nationalities, Baise, China

**Keywords:** colorectal cancer, serum lipid, systemic inflammation index, nomogram, prognosis

## Abstract

**Background:**

Lipid metabolism and cancer-related inflammation are closely related to the progression and prognosis of colorectal cancer (CRC). Therefore, this study aims to establish novel nomograms based on the combined detection of preoperative blood lipids and systemic inflammatory indicators to predict the overall survival (OS) and cancer-specific survival (CCS) of CRC patients.

**Methods:**

A total of 523 patients with stage I-III CRC in our institute were collected from 2014 to 2018. The independent predictors for OS and CCS were determined by forward stepwise Cox regression for the establishment of prognostic models. The superiorities of different models were compared by concordance index (C-index), Akaike information criterion (AIC) and integrated discrimination improvement analysis. The performance of the nomograms based on the optimal models was measured by the plotting time-dependent receiver operating characteristic curves, calibration curves, and decision curves, and compared with the tumor-node-metastasis (TNM) staging system. The cohort was categorized into low-risk, medium-risk and high-risk groups according to the risk points of the nomogram, and analyzed using Kaplan–Meier curves and log-rank test.

**Results:**

Preoperative TG/HDL-C ratio (THR) ≥ 1.93 and prognostic nutritional index (PNI) ≥ 42.55 were independently associated with favorable outcomes in CRC patients. Six (pT stage, pN stage, histological subtype, perineural invasion, THR and PNI) and seven (pT stage, pN stage, histological subtype, perineural invasion, gross appearance, THR and PNI) variables were chosen to develop the optimal models and construct nomograms for the prediction of OS and CCS. The models had lower AIC and larger C-indexes than other models lacking either or both of THR and PNI, and improved those integrated discrimination ability significantly. The nomograms showed better discrimination ability, calibration ability and clinical effectiveness than TNM system in predicting OS and CCS, and these results were reproducible in the validation cohort. The three risk stratifications based on the nomograms presented significant discrepancies in prognosis.

**Conclusion:**

Preoperative THR and PNI have distinct prognostic value in stage I-III CRC patients. The nomograms incorporated the two indexes provide an intuitive and reliable approach for predicting the prognosis and optimizing individualized therapy of non-metastatic CRC patients, which may be a complement to the TNM staging system.

## Introduction

Colorectal cancer (CRC) is one of the most common malignant tumors worldwide, and its incidence rate on the rise in recent years. It is reported that CRC is the cancer with the third highest incidence rate and the second highest mortality rate, which is second only to lung cancer (1). Surgical resection is currently the primary treatment for non-metastatic CRC, and about 50% of patients have recurrence or metastasis (2). Therefore, adjuvant therapy is recommended for CRC patients with high-risk factors. Despite continuous progress in treatment, the long-term survival rate of CRC patients is still not optimistic, with a 5-year survival rate of only 60% for those undergoing radical surgery (3).

At present, the criteria widely used to predict postoperative risk and develop treatment strategy of CRC patients is still the classification system of tumor-node-metastasis (TNM) approved by the American Joint Cancer Committee. Moreover, higher histological grading, positive lymphovascular invasion, positive perineural invasion, preoperative intestinal obstruction or perforation, elevated levels of preoperative carcinoembryonic antigen (CEA) and carbohydrate antigen 19-9 (CA19-9) have been generally recognized to be associated with recurrence, metastasis and short survival (4, 5). However, due to the heterogeneity of CRC, its clinical course is not always predictable, and patients with the same disease stage and similar pathological features may have different outcomes. Thus, it is always necessary for clinicians to identify new and effective factors related to the poor prognoses of CRC patients, so as to optimize personalized treatment.

A large number of studies have shown that lipid metabolism plays a key role in carcinogenesis and the invasive or metastatic procedure of neoplasms (6). For instance, abnormal triglyceride (TG) metabolism regulates tumor cell proliferation through adenosine monophosphate-activated protein kinase (AMPK) and mechanistic target of rapamycin (mTOR) pathway (7); cholesterol promotes tumor progression and chemotherapy resistance by altering cytoskeleton, angiogenesis and apoptosis (8); low-density lipoprotein cholesterol (LDL-C) promotes tumor progression through accumulating more reactive oxygen species and mitogen-activated protein kinase (MAPK) signaling pathway (9); and high-density lipoprotein cholesterol (HDL-C) may be associated with increased levels of anti-inflammatory cytokines such as interleukin (IL)-10, which reduces the production of pro-inflammatory cytokines such as IL-6 and tumor necrosis factor (TNF)-α, thereby inhibiting the growth and proliferation of tumor cells and promoting their apoptosis (10). It was reported that some routinely measured blood lipid parameters were effective prognostic factors for many solid malignant tumors, including CRC, prostate cancer, and breast cancer (11–13). In addition, serum lipid derivatives, including the ratio of TC minus HDL-C to HDL-C which is known as the atherosclerotic index (AI), the ratio of TG to HDL-C (THR), and the ratio of LDL-C to HDL-C (LHR), have been widely considered to be associated with cardiovascular and cerebrovascular diseases, and their prediction for prognosis is better than that of individual blood lipid indicators (14, 15). Previous studies have shown that serum lipid derivatives have significant predictive ability for postoperative survival of patients with malignant tumors such as breast and gastric cancer (16–18).

Tumor-related inflammatory response is closely related to tumor cell proliferation, angiogenesis, metastasis, anti-tumor immune disorder, and drug resistance of anti-cancer therapy (19). As markers of the systemic inflammatory, neutrophils and platelets secrete pro-inflammatory cytokines such as vascular endothelial growth factor, tumor necrosis factor, interleukin-2, and interleukin-6 to affect the development of tumors (20, 21). It is found that monocytes and lymphocytes play an anti-tumor role by enhancing the immune response to neoplasms (22, 23). Recently, some immune prognosis scores that can only be obtained by calculating the whole blood cell count and/or preoperative nutritional indicators, such as neutrophil/lymphocyte ratio (NLR) (24), platelet/lymphocyte ratio (PLR) (25), neutrophil/white blood cell ratio (NWR) (26), lymphocyte/monocyte ratio (LMR) (27), C-reactive protein (CRP)/albumin (Alb) ratio (CAR) (28), modified Glasgow prognostic score (mGPS) (29), systemic immune inflammation index (SII) (30) and prognostic nutritional index (PNI) (31, 32), have been proved to be able to predict the prognoses of a variety of malignant tumors including CRC.

Researchers have begun to pay attention to the subtle relationship between plasma lipids and inflammation in patients with malignancies. Blood lipids may affect the development of tumors by up-regulating or suppressing immune responses (11, 33). On the contrary, malignant tumors may also trigger low-grade acute phase response through systemic inflammatory responses, leading to changes in lipid metabolism (34). It is reasonable to propose a hypothesis that the combination of circulating serum lipids and immune indexes may be helpful to identify CRC patients with poor prognosis. In this study, we aim to explore the prognostic value of preoperative blood lipids and inflammatory indexes in patients with non-metastatic CRC undergoing radical surgery, and attempt to develop and validate novel and promising prognostic nomograms to complement TNM staging system and to optimize individualized prediction of such populations.

## Materials and methods

### Patient selection

**Figure 1:**
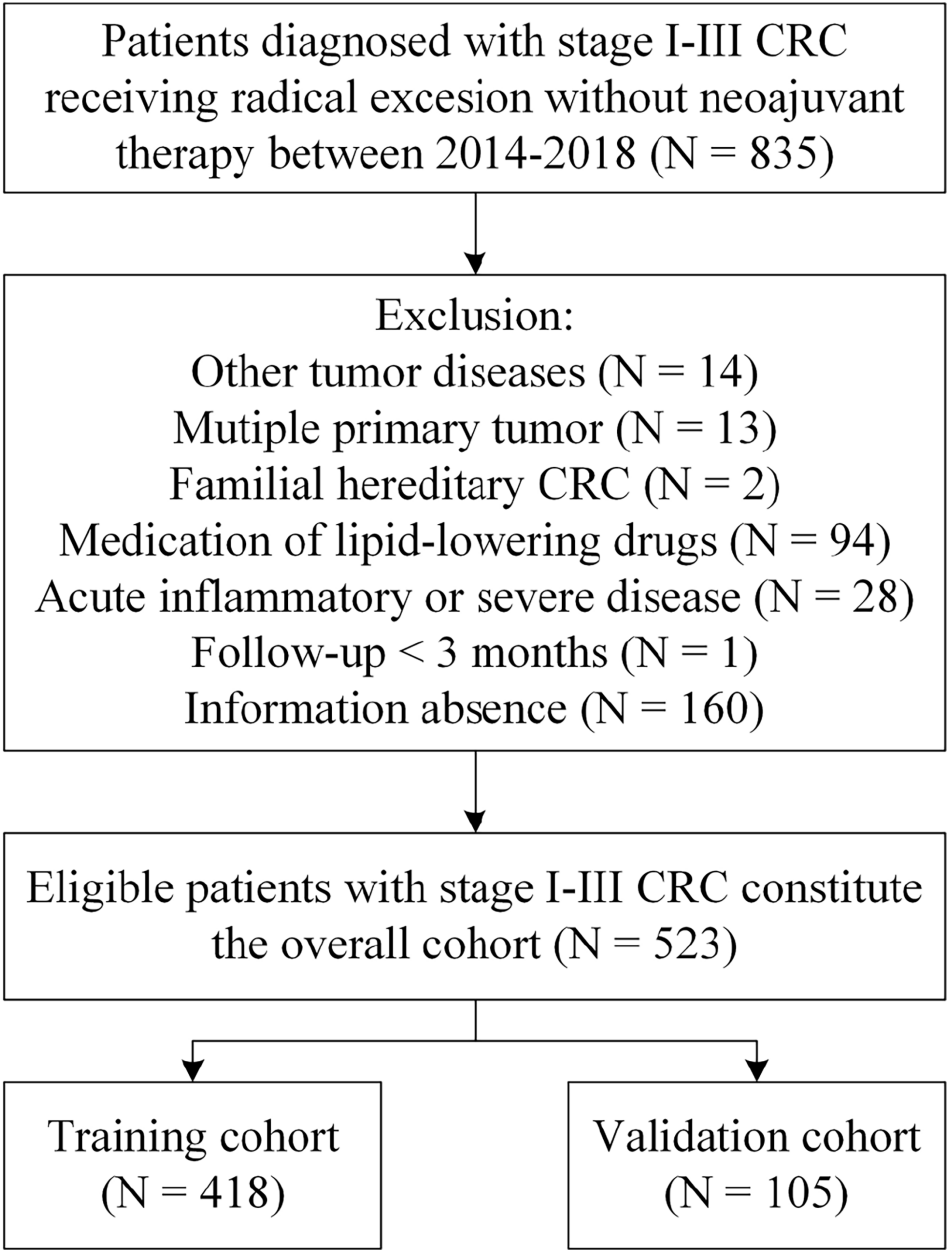
Flow diagram of patient selection.

We collected the medical records of CRC patients admitted to the Affiliated Tumor Hospital of Guangxi Medical University from January 2014 to December 2018. Patients who meet the following criteria will be included study: 1) patients who received radical surgery (surgical R0 excision) and were confirmed as CRC by histopathology; 2) without distant metastasis before operation; 3) patients did not receive any preoperative anti-tumor treatment. The exclusion criteria included: 1) patients who suffered from other malignant tumors in the past or at the same time; 2) patients with two or more primary tumors; 3) patients who diagnosed with familial hereditary CRC such as Lynch syndrome and familial adenomatous polyposis; 4) patients who have taken any drug known to affect blood lipids level, such as lipid-lowering drugs, glucocorticoids, and metformin within six months before collecting serum information; 5) patients who have any clinical evidence of acute infectious disease such as pneumonia and urinary tract infection, liver or kidney dysfunction, severe cardiovascular or cerebrovascular disease, and other serious diseases before surgery; 6) patients receiving less than 3 months of follow up; 7) patients lacking relatively complete and available clinicopathological, laboratory information and follow-up data. Eligible patients constitute the overall cohort (N = 523) and were randomly assigned to a training cohort (N = 418) or validation cohort (N = 105) in 4:1 ratio. The flow chart for cohort selection was described in **Figure 1**.

This study was approved by the Ethics Committee of the Affiliated Tumor Hospital of Guangxi Medical University (LW2023012). Individual consent from patients for this retrospective study was waived.

### Data collection and definition

All data were obtained from the electronic medical record system of the Affiliated Tumor Hospital of Guangxi Medical University. Patients’ clinical information included gender, age at diagnosis, body mass index (BMI), presence of preoperative intestinal obstruction or perforation, operation type, and the number of harvested lymph nodes in surgery. Among them, BMI was further divided into underweight (<18.5 kg/m^2^), normal weight (18.5-23.9 kg/m^2^), overweight (24.0-27.9 kg/m^2^) and obesity (≥28 kg/m^2^) in accordance with the diagnostic criteria in China (35). Tumor condition included TNM stage, pT stage, pN stage, histological subtype, differentiation degree, perineural invasion, lymphovascular invasion, tumor location, tumor size, and gross appearance. The pathological staging was performed according to the 8th edition of the American Joint Committee on Cancer (AJCC) TNM staging system.

Laboratory indicators included serum lipid indexes, inflammatory indexes, and tumor markers. All blood samples were obtained by drawing fasting venous blood from the participants within two weeks before surgery. Serum lipid indexes included total cholesterol (TC), TG, HDL-C, LDL-C, apolipoprotein A1 (apoA1), apolipoprotein B (apoB), lipoprotein(a) [Lp(a)], and three blood lipid derivatives such as AI, THR, and LHR. Among them, the levels of TC and TG were assayed with enzymatic methods, while HDL-C and LDL-C were detected with direct methods. Serum levels of apoA1, apoB, and Lp(a) were measured using immunoturbidimetry. The measurements were conducted on the Siemens ADVIA 2400 automatic biochemical analyzer, and the kits were purchased from commercial sources.

Inflammatory indexes included CAR, mGPS, SII, LMR, NLR, PLR, NWR, and PNI. Notably, mGPS was evaluated in light of the previously reported formula (36): score 0: no increase in CRP (≤ 10mg/L); score 1: increase in CRP (> 10mg/L), but normal level of Alb (≥ 35g/L); score 2: increase in CRP (> 10mg/L), and decrease in Alb (< 35g/L). SII and PNI were calculated according to the previously reported formulas (30, 37): SII = (platelet × neutrophil count)/lymphocyte count; PNI = 10 × serum Alb level (g/dL) + 0.005 × peripheral blood lymphocyte count (mm^3^). In addition, serum tumor markers such as CEA and CA19-9 were also collected.

### Follow-up

The Follow-up was performed every 3 to 6 months for the first 2 years after surgery, every 6 months for the next 3 years, and annually thereafter until patient’s death or March 2022. The primary endpoints were overall survival (OS) and tumor-specific survival (CCS). OS was defined as the interval from the date of surgery to death or the last follow-up, and CCS was defined as the interval from the date of surgery to death due to CRC or the last follow-up.

### Statistical analysis

Categorical variables were expressed as frequencies (percentages) and compared using the chi-square test or Fisher’s exact test. Cut-off values for tumor markers were defined according to conventional reference ranges. And for lipid and inflammatory markers, the optimal cut-off values were obtained in the overall cohort to predict OS by using the maximum x^2^ method in the R language “maxstat” package (38).

All variables were consistent with the proportional hazard assumption. Univariate Cox regression analysis was used to assess the impact of each variable on OS and CCS in the total cohort, and the variables with P < 0.10 were regarded as potential predictors. Multivariate Cox regression analysis with forward stepwise selection was performed to identify independent prognostic factors. First, we identified prognostic factors that independently predict OS and CCS among the clinicopathological variables, which were defined as basic risk factors. Then, these factors were entered into stepwise regression together with laboratory indicators for variable screening. All selected independent prognostic factors with P < 0.05 were used to establish predictive models for OS and CCS based on the training cohort. Meanwhile, in order to prove the superiorities of the models, a few models containing different combinations of independent candidate variables were constructed, such as blood lipid models including serum lipids and clinicopathological factors, inflammatory models including inflammatory indicators and clinicopathological factors, and basic risk models with only clinicopathological factors. Akaike information criterion (AIC) and concordance index (C-index) were used for model comparison. The smaller the AIC value and the larger the C-index, the better the model. In addition, the integrated discrimination improvement (IDI) was applied to measure the improvement in forecasting ability of models. Nomograms for the likelihood of OS and CCS at 3- and 5-year were developed separately based on the optimal models. The predictive performance of the nomograms was assessed in the training cohort and compared with the 8th AJCC TNM classification. Time-dependent receiver operating characteristic (ROC) curves were used to evaluate the predictive discrimination of the nomograms. Calibration curve and decision curve analysis were applied to assess the clinical consistency and effectiveness of the nomograms. The boot-strap resampling strategy was applied to validate the nomograms internally. Furthermore, the nomograms were evaluated utilizing the same method in the validation cohort. The differences in the survival curves of patients stratified into low-risk, medium-risk and high-risk categories according to the risk points calculated from the nomograms were analyzed by applying Kaplan-Meier (K-M) survival analysis and log-rank test.

All statistical analyses were performed by using SPSS software, version 25.0 (SPSS Inc., Chicago, IL, USA) and R 4.1.2 software (Institute of Statistics and Mathematics, Vienna, Austria). All tests were two-sided and P values less than 0.05 were considered statistically significant.

## Result

### Patient characteristics

A total of 523 CRC patients including 310 (59.3%) males and 213 (40.7%) females were included in this study. The median age at diagnosis was 60 (39, 68) years. Among them, lesions of 122 patients (23.3%) were located in the right colon, 145 patients (27.7%) were located in the left colon and 252 patients (48.2%) were located in the rectum. There were 109 (20.8%), 189 (36.1%), and 225 (43.0%) patients with TNM stage I, II, and III, respectively. The median follow-up time was 53 months, ranged from 3 to 97 months. The total cohort included 115 patients who died during the follow-up, of which 104 died of CRC and 11 died of other diseases. There was no statistically significant difference in baseline features between the training (N = 418) and validation cohorts (N = 105). Detailed characteristics in the cohorts were summarized in **Table 1**.

**Table 1:** Baseline characteristics.

Characteristics	Categories	Overall cohort (N = 523)	Training cohort (N = 418)	Validation cohort (N = 105)	P-value
Sex	Female	213 (40.7)	172 (41.1)	41 (39.0)	0.779
	Male	310 (59.3)	246 (58.9)	64 (61.0)	
Age (years)	< 60	251 (48.0)	199 (47.6)	52 (49.5)	0.809
	≥ 60	272 (52.0)	219 (52.4)	53 (50.5)	
BMI (kg/m^2^)	< 18.5	53 (10.1)	42 (10.0)	11 (10.5)	0.467
	18.5-23.9	334 (63.9)	268 (64.1)	66 (62.9)	
	24-27.9	111 (21.2)	91 (21.8)	20 (19.0)	
	≥ 28	25 (4.8)	17 (4.1)	8 (7.6)	
TNM stage	I	109 (20.8)	91 (21.8)	18 (17.1)	0.418
	II	189 (36.1)	146 (34.9)	43 (41.0)	
	III	225 (43.0)	181 (43.3)	44 (41.9)	
pT stage	T1/T2	134 (25.6)	113 (27.0)	21 (20.0)	0.126
	T3	150 (28.7)	123 (29.4)	27 (25.7)	
	T4	239 (45.7)	182 (43.5)	57 (54.3)	
pN stage	N0	300 (57.4)	238 (56.9)	62 (59.0)	0.880
	N1	155 (29.6)	126 (30.1)	29 (27.6)	
	N2	68 (13.0)	54 (12.9)	14 (13.3)	
Differentiation degree	High/moderate-high	56 (10.7)	45 (10.8)	11 (10.5)	0.270
	Moderate	393 (75.1)	319 (76.3)	74 (70.5)	
	Low/low-moderate	74 (14.1)	54 (12.9)	20 (19.0)	
Histological subtype	Non-mucinous	430 (82.2)	349 (83.5)	81 (77.1)	0.168
	Mucinous	93 (17.8)	69 (16.5)	24 (22.9)	
Perineural invasion	Negative	279 (53.3)	222 (53.1)	57 (54.3)	0.915
	Positive	244 (46.7)	196 (46.9)	48 (45.7)	
Lymphovascular invasion	Negative	373 (71.3)	299 (71.5)	74 (70.5)	0.926
	Positive	150 (28.7)	119 (28.5)	31 (29.5)	
Tumor location	Right-side colon	122 (23.3)	90 (21.5)	32 (30.5)	0.099
	Left-side colon	145 (27.7)	115 (27.5)	30 (28.6)	
	Rectum	256 (48.9)	213 (51.0)	43 (41.0)	
Tumor size (cm)	< 5cm	283 (54.1)	232 (55.5)	51 (48.6)	0.244
	≥ 5cm	240 (45.9)	186 (44.5)	54 (51.4)	
Gross appearance	Protruded	222 (42.4)	185 (44.3)	37 (35.2)	0.118
	Infiltrating/ulcerative	301 (57.6)	233 (55.7)	68 (64.8)	
Intestinal obstruction or perforation	No	512 (97.9)	411 (98.3)	101 (96.2)	0.326
	Yes	11 (2.1)	7 (1.7)	4 (3.8)	
Harvested lymph nodes (no.)	< 12	139 (26.6)	111 (26.6)	28 (26.7)	1.000
	≥ 12	384 (73.4)	307 (73.4)	77 (73.3)	
Operation type	Open surgery	90 (17.2)	77 (18.4)	13 (12.4)	0.186
	Laparoscopic surgery	433 (82.8)	341 (81.6)	92 (87.6)	
CEA (ng/mL)	≤ 5	354 (67.7)	289 (69.1)	65 (61.9)	0.194
	> 5	169 (32.3)	129 (30.9)	40 (38.1)	
CA19-9 (U/mL)	< 37	458 (87.6)	368 (88.0)	90 (85.7)	0.631
	≥ 37	65 (12.4)	50 (12.0)	15 (14.3)	
TC (mmol/L)	< 5.61	417 (79.7)	330 (78.9)	87 (82.9)	0.450
	≥ 5.61	106 (20.3)	88 (21.1)	18 (17.1)	
TG (mmol/L)	< 1.63	415 (79.3)	328 (78.5)	87 (82.9)	0.391
	≥ 1.63	108 (20.7)	90 (21.5)	18 (17.1)	
HDL-C (mmol/L)	< 1.47	439 (83.9)	351 (84.0)	88 (83.8)	1.000
	≥ 1.47	84 (16.1)	67 (16.0)	17 (16.2)	
LDL-C (mmol/L)	< 3.98	437 (83.6)	349 (83.5)	88 (83.8)	1.000
	≥ 3.98	86 (16.4)	69 (16.5)	17 (16.2)	
AI	< 4.49	455 (87.0)	362 (86.6)	93 (88.6)	0.708
	≥ 4.49	68 (13.0)	56 (13.4)	12 (11.4)	
THR	< 1.93	454 (86.8)	362 (86.6)	92 (87.6)	0.909
	≥ 1.93	69 (13.2)	56 (13.4)	13 (12.4)	
LHR	< 2.96	320 (61.2)	258 (61.7)	62 (59.0)	0.696
	≥ 2.96	203 (38.8)	160 (38.3)	43 (41.0)	
ApoA1 (g/L)	< 1.19	309 (59.1)	250 (59.8)	59 (56.2)	0.573
	≥ 1.19	214 (40.9)	168 (40.2)	46 (43.8)	
ApoB (g/L)	< 0.91	264 (50.5)	211 (50.5)	53 (50.5)	1.000
	≥ 0.91	259 (49.5)	207 (49.5)	52 (49.5)	
ApoA1/ApoB	< 0.89	69 (13.2)	54 (12.9)	15 (14.3)	0.835
	≥ 0.89	454 (86.8)	364 (87.1)	90 (85.7)	
Lpa (mg/L)	< 586.00	452 (86.4)	363 (86.8)	89 (84.8)	0.691
	≥ 586.00	71 (13.6)	55 (13.2)	16 (15.2)	
CAR	< 0.26	429 (82.0)	341 (81.6)	88 (83.8)	0.697
	≥ 0.26	94 (18.0)	77 (18.4)	17 (16.2)	
mGPS (Score)	0	435 (83.2)	346 (82.8)	89 (84.8)	0.474
	1	39 (7.5)	34 (8.1)	5 (4.8)	
	2	49 (9.4)	38 (9.1)	11 (10.5)	
SII	< 317.37	80 (15.3)	64 (15.3)	16 (15.2)	1.000
	≥ 317.37	443 (84.7)	354 (84.7)	89 (84.8)	
LMR	< 4.70	333 (63.7)	259 (62.0)	74 (70.5)	0.131
	≥ 4.70	190 (36.3)	159 (38.0)	31 (29.5)	
NLR	< 1.95	230 (44.0)	181 (43.3)	49 (46.7)	0.609
	≥ 1.95	293 (56.0)	237 (56.7)	56 (53.3)	
PLR	< 190.59	361 (69.0)	296 (70.8)	65 (61.9)	0.100
	≥ 190.59	162 (31.0)	122 (29.2)	40 (38.1)	
NWR	< 0.64	341 (65.2)	275 (65.8)	66 (62.9)	0.653
	≥ 0.64	182 (34.8)	143 (34.2)	39 (37.1)	
PNI	< 42.55	96 (18.4)	76 (18.2)	20 (19.0)	0.949
	≥ 42.55	427 (81.6)	342 (81.8)	85 (81.0)	

### Identification of basic risk factors for predicting OS and CCS

As classic prognostic factors, Clinicopathological variables such as pT stage, pN stage, gross appearance, differentiation degree, histological subtype, perineural invasion and lymphovascular invasion were all associated with the OS and CCS by univariate analysis in the overall cohort (all p < 0.10) (**Tables 2**, **3**). With further selection by forward stepwise Cox regression analysis, pT stage, pN stage, histological subtype, and Perineural invasion were determined as independent prognostic factors of OS (all p < 0.05) (**Table 2**), and pT stage, pN stage, gross appearance, histological subtype and perineural invasion were determined as independent predictors of CCS (all p < 0.05) (**Table 3**). All factors above were considered as the fundamental risk factors and will be used as the cornerstones for further variable screening.

**Table 2:** Univariate and multivariate Cox regression analysis of clinicopathological variables related to OS.

Variables	Univariate analysis		Multivariate analysis	
	HR (95% CI)	P-value	HR (95% CI)	P-value
Sex (Ref: female)	1.37 (0.93-2.01)	0.112		
Age (Ref: < 60 years)	1.05 (0.73-1.52)	0.780		
BMI (Ref: 18.5-23.9 kg/m^2^)		0.107		
< 18.5	1.82 (1.08-3.05)	0.024		
24.0-27.9	0.89 (0.55-1.45)	0.645		
≥ 28	1.14 (0.5-2.63)	0.752		
pT stage (Ref: T1/T2)		**< 0.001**		**0.004**
T3	4.57 (1.89-11.04)	0.001	3.20 (1.31-7.84)	0.011
T4	8.64 (3.77-19.80)	< 0.001	4.32(1.80-10.35)	0.001
pN stage (Ref: N0)		**< 0.001**		**< 0.001**
N1	2.36 (1.52-3.67)	< 0.001	1.72 (1.10-2.70)	0.018
N2	5.54 (3.51-8.74)	< 0.001	3.22 (2.00-5.18)	< 0.001
Differentiation degree (Ref: High/moderate-high)		**0.044**		
Moderate	1.21 (0.63-2.34)	0.568		
Low/low-moderate	2.07 (0.99-4.33)	0.053		
Histological subtype (Ref: Non-mucinous)	2.08 (1.39-3.11)	**< 0.001**	1.72 (1.14-2.58)	**0.010**
Perineural invasion (Ref: Negative)	2.90 (1.95-4.32)	**< 0.001**	1.74 (1.14-2.65)	**0.011**
Lymphovascular invasion (Ref: Negative)	2.43 (1.69-3.51)	**< 0.001**		
Location (Ref: Rectum)		0.500		
Right-side colon	0.79 (0.49-1.26)	0.318		
Left-side colon	0.82 (0.53-1.27)	0.373		
Tumor size (Ref: < 5cm)	1.16 (0.80-1.67)	0.439		
Gross appearance (Ref: Protruded type)	2.13 (1.42-3.21)	**< 0.001**		
Intestinal obstruction/perforation (Ref: No)	0.39 (0.06-2.82)	0.353		
Harvested lymph nodes (Ref: < 12 LNs)	0.99 (0.66-1.49)	0.960		
Operation type (Ref: Open surgery)	0.86 (0.55-1.37)	0.530		

P-value in bold font means statistically significant.

**Table 3:** Univariate and multivariate Cox regression analysis of clinicopathological variables related to CCS.

Variables	Univariate analysis		Multivariate analysis	
	HR (95% CI)	P-value	HR (95% CI)	P-value
Sex (Ref: female)	1.38 (0.92-2.08)	0.117		
Age (Ref: < 60 years)	0.90 (0.61-1.32)	0.584		
BMI (Ref: 18.5-23.9 kg/m^2^)		0.157		
< 18.5	1.86 (1.07-3.23)	0.027		
24-27.9	1.03 (0.62-1.68)	0.922		
≥ 28	1.31 (0.57-3.04)	0.523		
pT stage (Ref: T1/T2)		**< 0.001**		**0.040**
T3	5.09 (1.95-13.25)	0.001	2.81 (1.05-7.48)	0.039
T4	9.33 (3.77-23.08)	< 0.001	3.44 (1.31-9.02)	0.012
pN stage (Ref: N0)		**< 0.001**		**< 0.001**
N1	2.75 (1.71-4.41)	< 0.001	1.96 (1.22-3.17)	0.006
N2	6.39 (3.93-10.41)	< 0.001	3.36 (2.01-5.62)	< 0.001
Differentiation degree (Ref: High/moderate-high)		**0.007**		
Moderate	1.56 (0.72-3.39)	0.260		
Low/low-moderate	2.97 (1.28-6.89)	0.011		
Histological subtype (Ref: Non-mucinous)	1.90 (1.23-2.93)	**0.004**	1.68 (1.08-2.61)	0.021
Perineural invasion (Ref: Negative)	3.28 (2.14-5.04)	**< 0.001**	1.99 (1.26-3.14)	0.003
Lymphvascular invasion (Ref: Negative)	2.54 (1.73-3.73)	**< 0.001**		
Location (Ref: Rectum)		0.535		
Right-side colon	0.84 (0.51-1.36)	0.466		
Left-side colon	0.78 (0.49-1.25)	0.304		
Tumor size (Ref: < 5cm)	1.13 (0.77-1.66)	0.539		
Gross appearance (Ref: Protruded type)	2.60 (1.66-4.08)	**< 0.001**	1.75 (1.09-2.80)	**0.020**
Intestinal obstruction/perforation (Ref: No)	0.48 (0-17.25)	0.312		
Harvested lymph nodes (Ref: < 12 LNs)	0.99 (0.64-1.51)	0.947		
Operation type (Ref: Open surgery)	0.81 (0.50-1.29)	0.370		

P-value in bold font means statistically significant.

### Association between preoperative laboratory indicators and prognosis

In the entire cohort, univariate analysis showed that preoperative blood lipid indexes such as TC, HDL-C, LDL-C, THR, LHR, ApoA1, ApoB and Lp(a) were considered to be potential prognostic indicators for OS (all p < 0.10), while TC, TG, HDL-C, THR, LHR, ApoA1, and Lp(a) were potential prognostic indexes for CCS (all p < 0.10) (**Table 4**). Among the preoperative inflammatory indicators, univariate analysis showed that PNI was potentially associated with both OS and CCS (all p < 0.10) (**Table 4**). Besides, tumor markers such as CEA and CA19-9 also showed a correlation with prognosis in univariate analysis (**Table 4**). Further, both the identified basic risk factors and the candidate variables in preoperative laboratory indexes were collectively incorporated into the Cox regression analysis with stepwise forward. After multivariate analysis, only THR and PNI of blood indexes were finally selected (all p < 0.05) (**Table 5**). Our results showed that high THR level in patients was not only correlated with better OS (HR: 0.39, 95% CI: 0.19‐0.80, P = 0.010), but also correlated with better CCS (HR: 0.31, 95% CI: 0.14‐0.72, P = 0.006), and high PNI level in patients was associated with better OS and CCS (HR: 0.56, 95% CI: 0.36‐0.87, P = 0.010; HR: 0.50, 95% CI: 0.31‐0.81, P = 0.004, respectively) (**Table 5**).

**Table 4:** Univariate Cox regression analysis for OS and CCS in laboratory parameters.

Variables	OS		CCS	
	HR (95% CI)	P-value	HR (95% CI)	P-value
Tumor markers				
CEA (Ref: ≤ 5)	1.73 (1.19-2.50)	**0.004**	1.85 (1.26-2.72)	**0.002**
CA19-9 (Ref: < 37)	1.71 (1.07-2.75)	**0.026**	1.72 (1.04-2.82)	**0.034**
Serum lipid indexes				
TC (Ref: < 5.61)	0.52 (0.30-0.92)	**0.023**	0.59 (0.34-1.03)	**0.065**
TG (Ref: < 1.63)	0.67 (0.40-1.11)	0.116	0.61 (0.35-1.05)	**0.073**
HDL-C (Ref: < 1.47)	0.57 (0.31-1.04)	**0.065**	0.58 (0.31-1.08)	**0.086**
LDL-C (Ref: < 3.98)	0.61 (0.34-1.09)	**0.095**	0.63 (0.34-1.14)	0.128
AI (Ref: < 4.49)	0.72 (0.39-1.34)	0.300	0.81 (0.43-1.51)	0.501
THR (Ref: < 1.93)	0.46 (0.22-0.94)	**0.034**	0.38 (0.17-0.86)	**0.020**
LHR (Ref: < 2.96)	0.71 (0.48-1.06)	**0.090**	0.66 (0.44-1.01)	**0.056**
ApoA1 (Ref: < 1.19)	0.67 (0.45-0.98)	**0.041**	0.66 (0.44-0.99)	**0.044**
ApoB (Ref: < 0.91)	0.71 (0.49-1.03)	**0.068**	0.75 (0.51-1.11)	0.153
ApoA1/ApoB (Ref: < 0.89)	1.29 (0.71-2.35)	0.404	1.15 (0.63-2.10)	0.644
Lpa (Ref: < 586.00)	1.69 (1.06-2.69)	**0.027**	1.81 (1.12-2.92)	**0.015**
Inflammatory indexes				
CAR (Ref: < 0.26)	1.24 (0.78-1.95)	0.363	1.18 (0.72-1.92)	0.509
mGPS (Score 2 vs 1 vs 0)	1.06 (0.79-1.42)	0.712	1.00 (0.73-1.38)	0.982
LMR (Ref: < 4.70)	0.74 (0.50-1.10)	0.135	0.78 (0.52-1.19)	0.249
SII (Ref: < 317.37)	1.42 (0.80-2.52)	0.238	1.53 (0.82-2.85)	0.184
NLR (Ref: < 1.95)	1.30 (0.89-1.90)	0.168	1.19 (0.81-1.77)	0.377
PLR (Ref: < 190.59)	0.72 (0.47-1.09)	0.122	0.75 (0.48-1.16)	0.192
NWR (Ref: < 0.64)	1.22 (0.84-1.78)	0.291	1.10 (0.74-1.64)	0.650
PNI (Ref: < 42.55)	0.66 (0.43-1.02)	**0.060**	0.67 (0.43-1.07)	**0.091**

P-value in bold font means less than 0.10.

**Table 5:** Selected variables by multivariate forward stepwise Cox regression analysis.

Variables	Multivariate analysis	
	HR (95% CI)	P-value
OS		
pT stage (Ref: T1/T2)		**0.004**
T3	3.05 (1.24-7.49)	0.015
T4	4.25 (1.77-10.19)	0.001
pN stage (Ref: N0)		**< 0.001**
N1	1.84 (1.16-2.90)	0.009
N2	3.97 (2.43-6.50)	< 0.001
Histological subtype (Ref: Non-mucinous)	1.65 (1.10-2.48)	**0.016**
Perineural invasion (Ref: Negative)	1.64 (1.08-2.51)	**0.021**
THR (Ref: < 1.93)	0.40 (0.19-0.83)	**0.013**
PNI (Ref: < 42.55)	0.56 (0.36-0.87)	**0.011**
CCS		
pT stage (Ref: T1/T2)		**0.049**
T3	2.58 (0.96-6.91)	0.060
T4	3.26 (1.24-8.57)	0.016
pN stage (Ref: N0)		**< 0.001**
N1	2.12 (1.30-3.45)	0.002
N2	4.19 (2.47-7.11)	< 0.001
Histological subtype (Ref: Non-mucinous)	1.62 (1.04-2.52)	**0.032**
Perineural invasion (Ref: Negative)	1.86 (1.18-2.93)	**0.008**
Gross appearance (Ref: Protruded type)	1.90 (1.18-3.05)	**0.008**
THR (Ref: < 1.93)	0.32 (0.14-0.74)	**0.007**
PNI (Ref: < 42.55)	0.50 (0.31-0.81)	**0.005**

P-value in bold font means statistically significant.

### Development and comparison of novel prognostic models

According to the independent prognostic variables previously determined by multivariate regression, the blood lipid and inflammation models (model A and E), blood lipid models (model B and F), inflammation models (model C and G), and basic risk models (model D and H) were developed respectively to predict OS and CCS based on the training cohort (**Table 6**). Among them, model A predicting OS included pT stage, pN stage, histological subtype, perineural invasion, THR, and PNI, while model E predicting CCS included pT stage, pN stage, gross appearance, histological subtype, perineural invasion, THR, and PNI. In the comparison of different prognostic models (**Table 6**), the models with both THR and PNI had lower AIC values and higher C-indexes (with 1000 boot-strap resampling adjustments) (Model A: AIC: 1006.232; adjusted C-index: 0.741. Model E: AIC: 882.210; adjusted C-index: 0.763) than did models with other variables combination. And compared with the C-indexes of other models, the difference is statistically significant (all p < 0.05). The IDI analysis illustrated that the addition of PNI parameter could improve the integrated discrimination ability of blood lipid models (Model A vs model B: IDI = 0.027, p = 0.022; model E vs model F: IDI = 0.035, p = 0.022). Similarly, the predictive capability of inflammatory models was improved by adding THR parameter (Model A vs model C: IDI = 0.021, p = 0.018; model E vs model G: IDI = 0.026, p = 0.004). Compared with the basic risk model, the models combined with THR and PNI significantly improved comprehensive prediction ability (Model A vs model D: IDI = 0.050, p = 0.004; Model E vs model H: IDI = 0.063, p = < 0.001), especially more significant than the TNM staging models (Model A vs TNM: IDI = 0.116, p < 0.001; Model E vs TNM: IDI = 0.153, p < 0.001).

**Table 6:** Construction and comparison of different prognostic models.

Prognostic models	AIC	C-index		P-value^b^	IDI^c^	P-value^d^
		unadjusted	adjusted^a^			
OS						
Model A	1006.232	0.761	0.741	-	–	-
Model B	1012.556	0.746	0.728	**0.004**	0.027	**0.022**
Model C	1013.467	0.750	0.731	**0.002**	0.021	**0.018**
Model D	1020.590	0.733	0.716	**< 0.001**	0.050	**0.004**
TNM stage model	1039.352	0.661	0.660	**< 0.001**	0.116	**< 0.001**
CCS						
Model E	882.210	0.786	0.763	-	–	-
Model F	890.254	0.770	0.748	**0.002**	0.035	**0.022**
Model G	892.247	0.768	0.746	**< 0.001**	0.026	**0.004**
Model H	901.469	0.750	0.731	**< 0.001**	0.063	**< 0.001**
TNM stage model	922.730	0.670	0.669	**< 0.001**	0.153	**< 0.001**

Model A = pT stage + pN stage + Histological type + Perineural invasion + THR + PNI; Model B = pT stage + pN stage + Histological type + Perineural invasion + THR; Model C = pT stage + pN stage + Histological type + Perineural invasion + PNI; Model D = pT stage + pN stage + Histological type + Perineural invasion; Model E = pT stage + pN stage + Histological type + Perineural invasion + Gross appearance + THR + PNI; Model F = pT stage + pN stage + Histological type + Perineural invasion + Gross appearance + THR; Model G = pT stage + pN stage + Histological type + Perineural invasion + Gross appearance + PNI; Model H = pT stage + pN stage + Histological type + Perineural invasion + Gross appearance.

a C-index adjusted by boot-strap resampling strategy (1000 resamples).

b P-value of the likelihood-ratio test used to compare the C-index between model A, model E and other models in predicting OS and CCS, respectively.

c IDI analysis used to evaluate the improvement of model A and model E compared to other models in predicting OS and CCS, respectively.

d P-value of the IDI analysis. P-value in bold font means statistically significant.

### Construction and validation of novel nomograms

**Figure 2:**
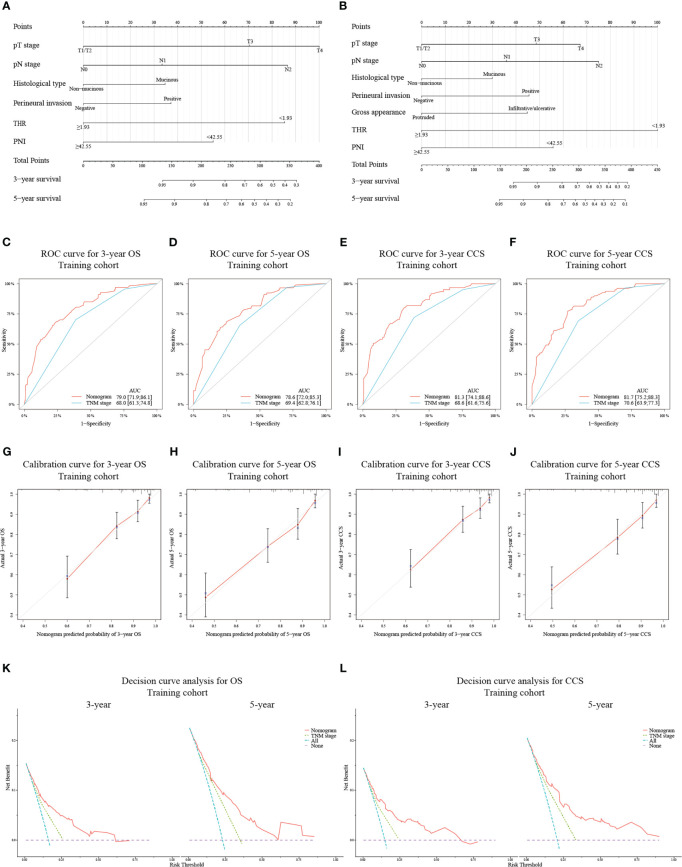
Construction of nomograms based on the training cohort to predict OS **(A)** and CCS **(B)** at 3- and 5-year in patients with non-metastatic CRC after receiving radical surgery. ROC curves for assessing the discrimination ability of the nomograms and TNM staging system for OS **(C, D)** at 3- and 5-year and for CCS **(E, F)** at 3- and 5-year in the training cohort. Calibration curves for evaluating the clinical consistency of the nomograms in predicting 3- and 5-year OS **(G, H)** and 3- and 5-year CCS **(I, J)** in the training cohort. Decision curves of the novel nomograms and TNM classification for predicting 3- and 5-year survival of OS **(K)** and CCS **(L)** in the training cohort.

Novel nomograms were constructed on the strength of the optimal models (**Figures 2A, B**). The predicted area under the curve (AUC) values for 3- and 5-year OS and CCS in the training cohort utilizing the nomograms were 79.0 (95% CI: 71.9-86.1) and 78.6 (95% CI: 72.0-85.3) (**Figures 2C, D**), and 81.3 (95% CI: 74.1-88.6) and 81.7 (95% CI: 75.2-88.3) (**Figures 2E, F**), respectively, all of which were superior to the AUC values predicted by TNM stage. The calibration curve adjusted by 1000 times boot-strap resampling also indicated that the prediction probability of the nomograms for 3- and 5-year OS and CCS were consistent with the actual observation (**Figures 2G–J**). Finally, we draw decision curves to illustrate the clinical applicability of the nomograms. The decision curves showed that the clinical effectiveness of the nomograms is better than that of TNM staging system within the actual threshold probability range (**Figures 2K, L**).

**Figure 3:**
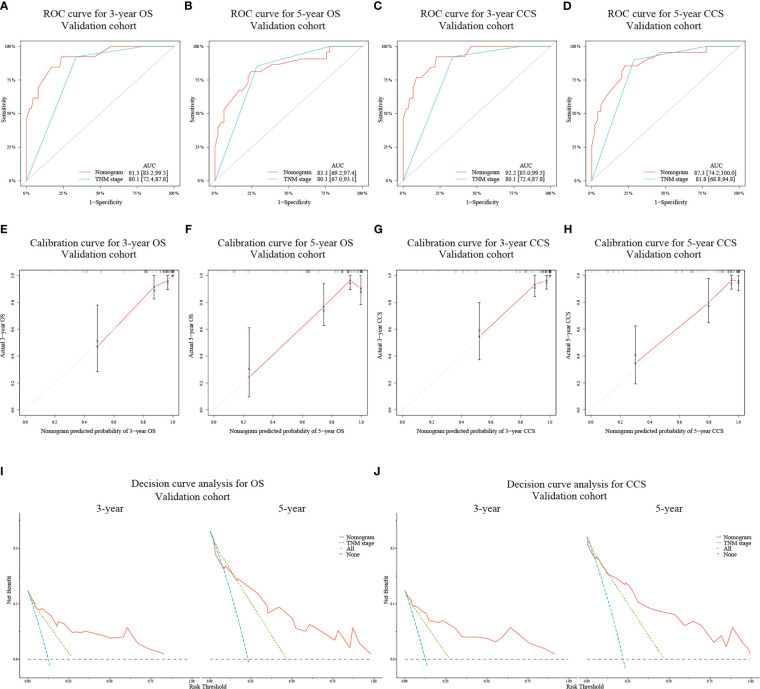
ROC curves for assessing the discrimination ability of the nomograms and TNM staging system for OS **(A, B)** at 3- and 5-year and for CCS **(C, D)** at 3- and 5-year in the validation cohort. Calibration curves for evaluating the clinical consistency of the nomograms in predicting 3- and 5-year OS **(E, F)** and 3- and 5-year CCS **(G, H)** in the validation cohort. Decision curves of the novel nomograms and TNM classification for predicting 3- and 5-year survival of OS **(I)** and CCS **(J)** in the validation cohort.

In the validation cohort, the AUC values for predicting 3- and 5-year OS and CCS using the nomograms were 91.3 (95% CI: 83.2-99.5) and 83.3 (95% CI: 69.2-97.4) (**Figures 3A, B**), and 92.2 (95% CI: 85.0-99.5) and 87.3 (95% CI: 74.2-100.0) (**Figures 3C, D**), respectively, all of which were also higher than those of TNM stage. Similarly, the calibration curves (**Figures 3E–H**) and decision curves (**Figures 3I, J**) showed that the nomograms had favorable calibration capacity and clinical efficacy in predicting 3- and 5-year OS and CCS in the validation set.

**Figure 4:**
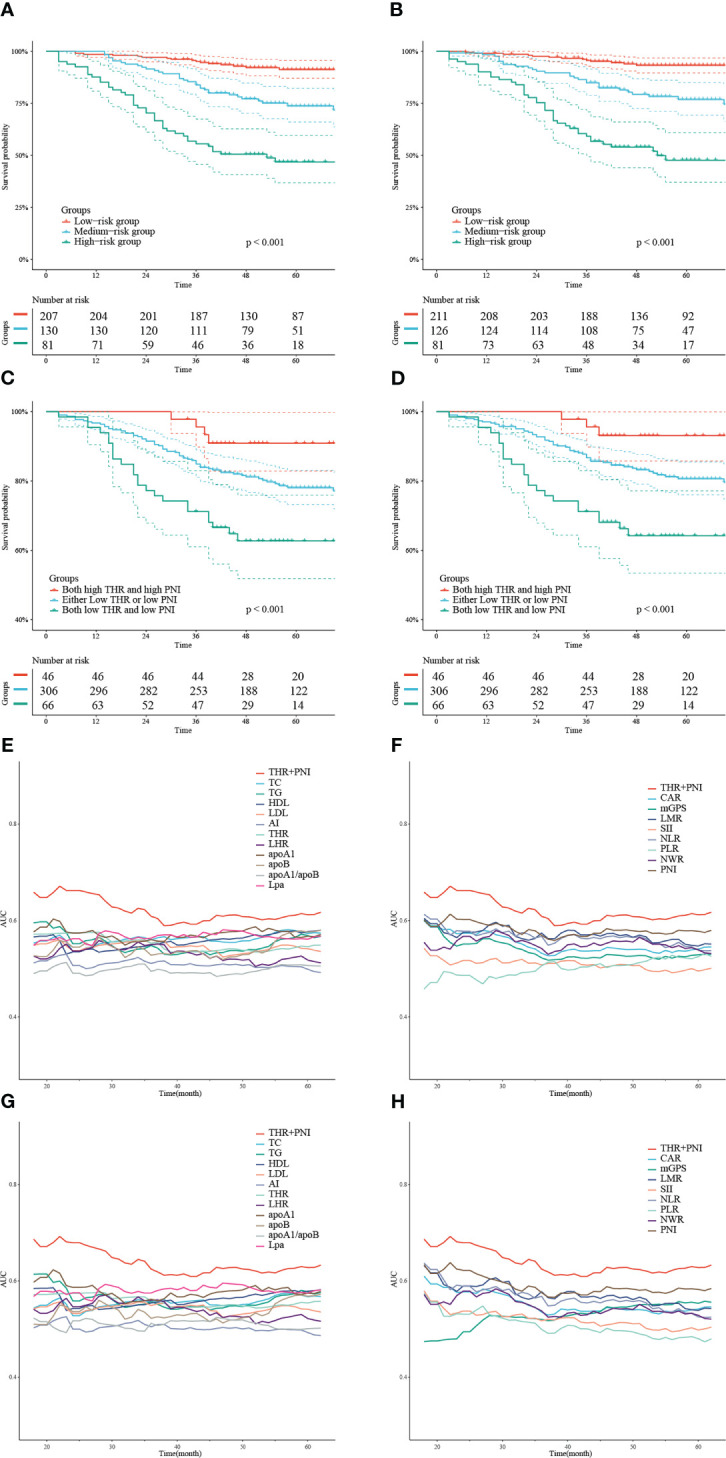
K-M curves depicting OS **(A)** and CCS **(B)** of the training cohort stratified by 50% and 80% percentiles of risk points calculated based on the nomograms. K-M survival curves describing OS **(C)** and CCS **(D)** of the training cohort categorized into 3 groups according to the combination of THR and PNI at different levels. Time-dependent AUC curves showing the AUC values of THR combined with PNI compared to serum lipid or systemic inflammatory indexes alone to predict OS **(E, F)** and CCS **(G, H)**.

According to the gross risk score assigned to each patient by nomograms, the cases in the training cohort were ranked in ascending order and divided into low-risk, medium-risk, and high-risk groups with 50% and 80% percentiles as the cut-off values (For OS: 190.48, 260.99; for CCS: 212.01, 287.03). The K-M survival curves revealed that the differences of survival rate among the groups were statistically significant (all p < 0.001) (**Figures 4A, B**).

### The prognostic value of combined THR and PNI in patients with CRC following radical surgery

We divided the training cohort into three groups: both high THR and high PNI, either low THR or low PNI, and both low THR and low PNI. The K-M survival curves showed that patients with low THR and low PNI had the shortest OS and CCS (all p < 0.05) (**Figures 4C, D**). In addition, we also plotted time-dependent AUC curves of each indicator. The results showed that the combination of THR and PNI could achieve higher AUC values in predicting OS and CCS during 20-60 months than using blood lipid or inflammatory parameters alone (**Figures 4E–H**).

### The relationship between THR and PNI and clinicopathological characteristics

To better understand the role of THR and PNI in CRC prognosis, we further analyzed the correlation between them and clinicopathological features in the entire cohort (**Table 7**). Patients with higher BMI and tumor located in the left colon or rectum had higher THR levels, indicating that THR level may be influenced by BMI and tumor location. Patients with higher PNI level tended to have younger age, have more advanced pN stage, have a higher proportion of in the left colon or rectum tumors, and have smaller tumor. These results suggested that the PNI level may be influenced by the age of patient, pN stage of the tumor, tumor location, and tumor size.

**Table 7:** Correlations between serum THR, PNI and clinicopathological factors in the overall cohort.

Variables	THR			PNI		
	< 1.93	≥ 1.93	P-value	< 42.55	≥ 42.55	P-value
	(N = 454)	(N = 69)		(N = 96)	(N = 427)	
Sex						
female	188 (41.41)	25 (36.23)	0.494	37 (38.54)	176 (41.22)	0.713
male	266 (58.59)	44 (63.77)		59 (61.46)	251 (58.78)	
Age						
<60	213 (46.92)	38 (55.07)	0.257	27 (28.12)	224 (52.46)	**< 0.001**
≥60	241 (53.08)	31 (44.93)		69 (71.88)	203 (47.54)	
BMI						
< 18.5	51 (11.23)	2 (2.90)	**0.009**	15 (15.62)	38 (8.90)	0.067
18.5-23.9	295 (64.98)	39 (56.52)		64 (66.67)	270 (63.23)	
24-27.9	89 (19.60)	22 (31.88)		15 (15.62)	96 (22.48)	
≥ 28	19 (4.19)	6 (8.70)		2 (2.08)	23 (5.39)	
pT stage						
T1	9 (1.98)	1 (1.45)	0.723	1 (1.04)	9 (2.11)	0.252
T2	111 (24.45)	13 (18.84)		16 (16.67)	108 (25.29)	
T3	130 (28.63)	20 (28.99)		32 (33.33)	118 (27.63)	
T4	204 (44.93)	35 (50.72)		47 (48.96)	192 (44.96)	
pN stage						
N0	258 (56.83)	42 (60.87)	0.100	65 (67.71)	235 (55.04)	**0.033**
N1	141 (31.06)	14 (20.29)		25 (26.04)	130 (30.44)	
N2	55 (12.11)	13 (18.84)		6 (6.25)	62 (14.52)	
Differentiation degree						
High/moderate-high	49 (10.79)	7 (10.14)	0.985	11 (11.46)	45 (10.54)	0.495
Moderate	341 (75.11)	52 (75.36)		68 (70.83)	325 (76.11)	
Low/low-moderate	64 (14.10)	10 (14.49)		17 (17.71)	57 (13.35)	
Histological subtype						
Non-mucinous	371 (81.72)	59 (85.51)	0.550	78 (81.25)	352 (82.44)	0.899
Mucinous	83 (18.28)	10 (14.49)		18 (18.75)	75 (17.56)	
Perineural invasion						
Negative	238 (52.42)	41 (59.42)	0.339	47 (48.96)	232 (54.33)	0.401
Positive	216 (47.58)	28 (40.58)		49 (51.04)	195 (45.67)	
Lymphvascular invasion						
Negative	324 (71.37)	49 (71.01)	1.000	67 (69.79)	306 (71.66)	0.809
Positive	130 (28.63)	20 (28.99)		29 (30.21)	121 (28.34)	
Location						
Right-side colon	116 (25.55)	6 (8.70)	**0.005**	38 (39.58)	84 (19.67)	**< 0.001**
Left-side colon	119 (26.21)	26 (37.68)		28 (29.17)	117 (27.40)	
Rctum	219 (48.24)	37 (53.62)		30 (31.25)	226 (52.93)	
Tumor size						
< 5cm	246 (54.19)	37 (53.62)	1.000	26 (27.08)	257 (60.19)	**< 0.001**
≥ 5cm	208 (45.81)	32 (46.38)		70 (72.92)	170 (39.81)	
Gross appearance						
Protruded type	198 (43.61)	24 (34.78)	0.211	49 (51.04)	173 (40.52)	0.077
Infiltrating/ulcerative type	256 (56.39)	45 (65.22)		47 (48.96)	254 (59.48)	

P-value in bold font means statistically significant.

## Discussion

Based on the single center retrospective cohort data, we investigated the effects of clinicopathological factors, blood lipid indexes, and systemic inflammatory indexes on the prognoses of non-metastatic CRC patients undergoing curative excision. Through multivariate analysis with forward stepwise, novel nomograms were established according to the optimal models containing THR and PNI, which could effectively predict the OS and CCS of the population at 3- and 5-year. Compared with traditional TNM classified system, nomograms showed better differentiation, accuracy and clinical applicability.

Given the role of lipid metabolism in carcinogenesis and the invasive or metastatic procedure in CRC, researchers are always keen to develop lipid parameters as new and convenient biomarkers for prognoses. THR is one of the derivatives of blood lipids. Previous studies proposed that its high level is associated with poor postoperative prognosis of breast cancer and gastric cancer (16, 17). However, there are still few studies on the predictive role of three lipid derivatives including THR in CRC patients. This study excluded people with non-cancerous factors that may affect blood lipid level, and adjusted other variables, indicating that higher THR level is independently associated with reduced risk of death. As far as we know, this study is the first time to propose THR as a meaningful prognostic marker for non-metastatic CRC patients. The contradictory assessment of the prognostic role of THR may be caused by different tumor types, study populations, and cut-off values. Although it remains unclear why a higher THR level is associated with a better prognosis of CRC, several speculations may explain this phenomenon. Firstly, it is speculated that such association is related to the higher level of TG and the corresponding lower HDL-C concentration. In fact, the existing studies on the relationship between these two lipid indexes and the prognoses of CRC is not sufficient, and the results are inconsistent. Yang et al. reported that dyslipidemia, including high serum level of TG and low level of HDL-C, was independently associated with the improvements of OS and recurrence-free survival in patients with colon cancer (12). Yin et al. found that increased adipose triglyceride lipase is negatively correlated with the OS of CRC patients, and *in vivo* experiments showed that it could promote the progression of CRC by enhancing lipid mobilization or lipolysis (40), which also reflects that high serum TG level are related to the improvement of prognosis. Other studies showed that increased TG or decreased HDL-C are associated with poor prognosis in CRC (11, 41), or not (13, 42). However, although some studies seem to support THR as an independent protective factor for CRC patients, further exploration is needed. Secondly, in the correlation analysis between THR and clinicopathological factors, we found that the high BMI was significantly related to the high levels of THR. Since TG is one of the prime lipid metabolites involved in energy supply, our findings support the hypothesis that an elevated level of THR may be mainly driven by TG concentration, which represent a better nutritional status and is related to a good prognosis of CRC. Thirdly, we also found that THR level in patients with tumors located in the right colon are significantly lower than those in the left colon or rectum. Despite no statistical significance has been observed in predicting survival in our study, previous studies have shown that the prognoses of patients with left-sided neoplasms are better than those of patients with right-sided neoplasms (43). It will be meaningful to further explore how the pathways affected by blood lipid profile interact with the carcinogenic pathways of different site (44).

Although indexes of systemic inflammation have been reported to predict cancer prognosis in recent years, it is still uncertain which marker has the greatest clinical application value. In this study, PNI was screened to be an independent protective factor affecting the postoperative survival of non-metastatic CRC patients by stepwise forward multivariate analysis. Onodera et al. firstly calculated PNI based on serum Alb level and peripheral blood lymphocyte count (37), and a large number of studies have confirmed its prognostic value in various cancers (31, 32). The activation of immune response can promote the protective response of cancer patients, which mainly depends on the levels of lymphocytes. A possible mechanism is that circulating lymphocytes may promote cytotoxic cell death to exert anti-tumor effect by secreting cytokines such as interferon-gamma and tumor necrosis factor-alpha (23). Alb, another component of PNI, is a common index to evaluate nutritional status in clinic. Malnutrition can inhibit the immune response by regulating the production of some cytokines and hormones which mainly affect T-lymphocytes metabolism and function (45). Moreover, poor nutritional status may delay surgery or adjuvant treatment for patients. However, there was also evidence that hypoproteinemia in CRC patients is associated with serum Alb degradation caused by systemic inflammatory response during tumor progression, rather than reduced synthesis caused by malnutrition alone (46). Therefore, low preoperative Alb level is usually associated with poor prognosis in patients with solid tumors. In summary, increased PNI levels may indicate that patients have a valid protective immune response and better nutritional status, so as to achieve longer survival.

Some studies have revealed the potential relationship between blood lipid derivatives and systemic inflammation. Blood lipid derivatives could serve as surrogate biomarkers of insulin resistance (IR) which is identified as a chronic subclinical inflammation in various chronic diseases including cancer (47, 48). The immune pathway mediated by pro-inflammatory cytokines such as IL-6 and TNF-α interferes with the biological effects of the insulin receptor downstream signaling and results in IR (49), which is also found to be closely involved in cancer development (50). Chronic IR is present in malignancies, and is speculated to contribute to tumor-related cachexia due to chronic exposure of pro-inflammatory cytokines and insulin growth factor binding protein (51). Because of the close interaction between IR and systemic inflammation in cancer patients, serum lipid derivatives combined with systemic inflammatory indicators may have an important predictive effect on cancer prognosis, as highlighted by a multicenter prospective study from China (18).

To our knowledge, THR and PNI were first applied together as independent prognostic factors for patients with non-metastatic CRC. While the combination of THR and PNI showed relatively higher AUC values and better predictive ability compared to using serum lipid or inflammatory indicators alone in predicting OS and CCS, it should be noted that the AUC values of the combined use of these two markers did not reach the clinically recommended level, which may contribute to their limited application in clinical practice. In our study, the combined detection of THR and PNI could help to screen patients with high risk of death, i.e. patients with both low THR and low PNI display the worst postoperative survival, indicating that there may be a synergistic effect between the two indexes in predicting CRC prognosis. Moreover, compared with the models including other combinations of variables or the TNM staging model, the models containing THR and PNI showed greater superiorities in the comparison of AIC and C-index. IDI analysis also revealed that the performance of the new prognostic models was significantly improved with the addition of THR and PNI. As discussed above, the combination of these two indicators may reflect a tumor-related metabolic and inflammatory state of the host, which could provide additional information for prognostic prediction of cancer. Although external data validation is still needed to confirm our findings, we believe that incorporating THR and PNI with traditional clinicopathologic features such as established TNM stage into an integrated system may lead to a more comprehensive and accurate prediction of survival outcomes for CRC patients.

In view of tumor heterogeneity and individual differences in nutrition and metabolism, there is no clear cut-off point for serum lipids and systematic inflammatory indicators to predict the prognosis of cancer patients. In this study, we used the maximum x^2^ method to obtain the optimal cut-off value of the above indicators, which could divide the cohort into two groups with maximum discrepancy based on log-rank statistics. Compared with the cut-off values obtained by arbitrary number method, median value method or ROC curves, our method could appropriately reflect the correlation between binary independent variables and dependent variables in time-to-event data. It is worth noting that the cut-off values of THR and PNI in this study were 1.93 and 42.55, respectively. Several reports (31, 39, 52, 53) on CRC utilized ROC curves analysis or classification and regression tree analysis to determine the best threshold value of preoperative PNI as 42.4 or higher, and found that the high-level group was associated with the lower incidence of postoperative complications and improved prognosis, which was consistent with our results. Nevertheless, there is still no large-scale cohort evidence to determine the cut-off value of THR for predicting postoperative survival of CRC patients, and further exploration is needed to facilitate clinical promotion and application.

The nomograms in this study contained pathological prognostic factors that have been widely recognized and utilized, such as pT stage, pN stage, histological subtype, and Perineural invasion. Furthermore, it is worth mentioning that gross morphology of tumors was identified as an independent candidate for predicting the clinical outcomes of stage I-III CRC patients undergoing radical operation. This is consistent with previous study which showed that patients with protruded type CRC have a lower risk of cancer-specific death (54). As a parameter that could be obtained directly by endoscopy or surgery, the predictive role of macroscopic morphology of CRC should not be underestimated.

The present research has several merits. First of all, through the detailed review of electronic medical records, the interference of non-CRC factors on blood lipids and inflammatory parameters was excluded with stricter criteria, which made the prognostic significance of above indexes more convincing and the models more robust. On the other hand, compared with some models based on large sample data obtained from the Surveillance, Epidemiology, and End Result (SEER) database, although the number of cases enrolled in this study is comparatively finite, we have obtained more detailed laboratory indicators than those found in tumor registration. Finally, our nomogram contains risk factors that could be easily collected from clinical practice. Easy accessibility, low cost and clinical applicability are prospects of the nomograms.

This study still has some limitations: 1) A retrospective study based on a single-center samples only, which may lead to selection and memory bias; 2) Lack of diet and lifestyle information of the surveyed population, which may affect the measurement of preoperative blood lipids and lead to potential deviations; 3) We only analyzed the relationship between preoperative THR and PNI and prognosis, and failed to monitor their dynamic variation in the disease process, which need to be further explained; 4) Given the screening conditions of this study, the application scenarios of constructed nomograms are limited; 5) The nomograms were only internally validated using data from a single center, and its generalizability needs further external data validation. Therefore, large-scale, multicenter prospective research is still needed in the future.

## Conclusion

Our study suggests that preoperative THR and PNI are independent predictors for survival of patients with stage I-III CRC. We have successfully established and verified the novel nomograms integrating preoperative THR and PNI, which will help clinicians to conveniently and accurately evaluate the prognosis of these patients and identify high-risk groups, so as to formulate individualized therapeutic regimens and follow-up strategies in time.

## Data availability statement

The raw data supporting the conclusions of this article will be made available by the authors, without undue reservation.

## Ethics statement

This study was approved by the Ethics Committee of the Affiliated Tumor Hospital of Guangxi Medical University (LW2023012). Individual consent from patients for this retrospective study was waived.

## Author contributions

DH designed the study, performed the statistical analysis, and wrote the manuscript. SZ participated in the manuscript revision. FH, JC, YZ and YC participated in the clinical data collection and assembly. BL conceived of the research and engaged in the supervision and critical review. All authors contributed to the article and approved the submitted version.
